# Case Report: A familial hematological pedigree reveals VHL germline mutation as a principal predisposition factor with additional mutations modulating phenotypic heterogeneity

**DOI:** 10.3389/fonc.2025.1630715

**Published:** 2025-07-29

**Authors:** HuiLing Chen, Wanli Hu, Chengcheng Ma, Miaomiao Zhang, Fuhua Yang, Pengyun Zeng

**Affiliations:** ^1^ Department of Hematology, Lanzhou University Second Hospital, Lanzhou, Gansu, China; ^2^ The Second Clinical Medicine School, Lanzhou University, Lanzhou, Gansu, China

**Keywords:** VHL, ASXL3, CCR7, germline mutation, familial hematological disease, genetic

## Abstract

**Background:**

VHL germline mutations are classically associated with von Hippel-Lindau syndrome, but their role in hematological malignancies remains underexplored.

**Methods:**

We analyzed a pedigree with acute myeloid leukemia (AML) proband and two offspring: primary immune thrombocytopenia (ITP) and acute T-cell lymphoblastic leukemia (T-ALL) via targeted sequencing and familial validation.

**Results:**

Genetic analysis revealed: (1) the proband carried concurrent VHL, ASXL3, and CCR7 germline mutations along with acquired BCOR/NF1 variants; (2) the ITP-affected offspring inherited ASXL3/CCR7 mutations only; and (3) the T-ALL case exhibited solely the VHL mutation. Acquired mutations (e.g., BCOR/NF1) in the proband suggest a ‘two-hit’ model for leukemogenesis.

**Conclusion:**

This study identifies VHL as the principal predisposing mutation in a familial hematologic malignancy pedigree presenting with heterogeneous phenotypes, where ASXL3/CCR7 variants may serve as phenotypic modifiers. These findings advocate for genotype-driven surveillance strategies in familial hematological disorders.

Germline mutations can lead to the development of familial and sporadic hematological disorder, including acute lymphoblastic leukemia (ALL), acute myeloid leukemia (AML) and myelodysplastic syndrome (MDS) (1). Genetic heterogeneity often stems from the cumulative effects of germline mutations, but clinical reports of multigene synergistic pathogenesis remain exceedingly rare. VHL, a classic tumor suppressor gene, is commonly associated with germline mutations in von Hippel-Lindau syndrome. However, its involvement in hematological malignancies is scarcely documented (2, 3). ASXL family members are epigenetic scaffolding proteins that assumed to be tumor suppressive or oncogenic. Truncation mutations of ASXL1 occur in malignant myeloid diseases, and ASXL3 is first identified as a distinct neurodevelopmental disorder, but its role in myeloid/lymphoid cell differentiation remains unclear (4). CCR7 encodes a chemokine receptor critical for lymphocyte migration, and its variants may be linked to immune microenvironment dysregulation (5). To date, no literature has reported a familial hematological pedigree with coexisting germline mutations in VHL, ASXL3 and CCR7. This study aims to characterize the synergistic effects of VHL/ASXL3/CCR7 germline mutations in a familial hematological pedigree.

## Case report

### Proband (patient 1)

A 45-year-old male presented in July 2023 with intermittent left-sided headache. Physical examination revealed no lymphadenopathy or hepatosplenomegaly.

#### Laboratory findings

**Complete Blood Count (CBC)**: WBC 4.2×10^9^/L, Hb 79g/L, PLT 13×10^9^/L.

Bone Marrow Morphology: Hypercellularity with 45% blasts and immature granulocytes.

**Immunophenotyping**: The flow cytometry revealed a myeloid blast population (40.8%) expressing CD34, CD117, CD13 and partially expressing CD33/CD38/CD45, while lacking lymphoid markers (CD7/CD10/CD19/CD20/CD2/CD3/CD4/CD8) and monocytic/granulocytic markers (CD14/CD15/CD11B/CD64/CD56), accompanied by an abnormal granulocytic population (33.7%) with diminished CD15 and aberrant CD13/CD33 expression patterns.

**Karyotype:** 46, XY, del(5)(q13q34)[4]/46, XY[6].

**Molecular Genetics:**

**Germline Mutations**: ASXL3 c.4816C>T (p.Arg1606Trp) (VAF 49.8%), CCR7 c.316C>G (p.Leu106Val) (VAF 18.7%), VHL c.385C>G (p.Leu129Val) (VAF 48.8%).

**Acquired Mutations**: BCOR p.Arg1164Ter (VAF 93.1%), NF1 compound mutations (VAF 2.3%-62.1%), NRAS p.Gly12Cys (VAF 5.8%), GATA2 p.Ser359Ala (VAF 43.7%).

**Diagnosis**: Flow cytometry showed aberrant myeloid antigen expression, consistent with acute myeloid leukemia with myelodysplasia-related gene mutations (AML-MR).

#### Treatment and outcome

**Induction**: Venetoclax+IA (venetoclax 100 mg, po, day 1; 200 mg, day 2, 400 mg days 1-7, idarubicin 12 mg/m² days 1-2, cytarabine 100 mg/m² days 1-5) induction achieved CR (blasts <5%) with persistent MRD (2.87%).

**Consolidation**: The patient completed 5 cycles of consolidation therapy (3 cycles high-dose cytarabine: 3 g/m² q12h, days 1-3, one cycle each of HA: homoharringtonine 2 mg/m²/day days 1-7, cytarabine 100 mg/m²/day days 1–7 and DA: daunorubicin 45 mg/m²/day days 1-3, cytarabine 100 mg/m²/day days 1-7) with sustained morphological complete remission with partial hematologic recovery (CRp) and MRD-negative status throughout.

**HSCT Rationale**: Allo-HSCT was deferred due to persistent thrombocytopenia (PLT <40×10^9^/L post-consolidation) and lack of a matched donor.

**Maintenance with venetoclax/azacitidine** was prioritized given ASXL3/BCOR alterations, which preclinical studies associate with hypomethylating agent sensitivity.

**Follow-Up**: As of the last follow-up in April 2025, the patient remains alive with stable disease control.

### Patient 2 (eldest son)

A 24-year-old male diagnosed with immune thrombocytopenic purpura (ITP) at age 10 (2011) due to petechiae.

#### Initial features

##### Laboratory findings

CBC: PLT 15×10^9^/L (normal WBC/Hb).

Bone marrow: Megakaryocytic maturation arrest.

**Exclusion workup:** Negative ANA, anti-platelet antibodies; normal coagulation.

**Diagnosis:** Primary immune thrombocytopenia (ITP).

#### Treatment

**First-line:** Initial methylprednisolone therapy (1 mg/kg/day×4 weeks) transiently increased platelets to 85×10^9^/L, but levels fell to 20-30×10^9^/L post-tapering, establishing steroid dependence.

**Molecular Findings** (2023 Evaluation):

Germline Variants: ASXL3 c.4816C>T (p.Arg1606Trp) (VAF 52.1%), CCR7 c.316C>G (p.Leu106Val) (VAF 47.3%),VHL c.385C>G (p.Leu129Val)(VAF 32.5%).

Somatic Alterations: BCOR p.Gln404Ter (VAF 7.1%), BCORL1 p.Pro1010Arg (VAF 99.9%).

**Second-line Therapy:**

Recombinant thrombopoietin (15,000 U/day×14 days, suboptimal response) and thrombopoietin receptor agonists (eltrbombopag 25–50 mg/day) have maintained platelets at 20-40×10^9^/L, with current continuation on eltrombopag.

**Current Status (2025):**

PLT 20-40×10^9^/L (asymptomatic), no progression to myelodysplastic syndromes or leukemia.

### Patient 3 (younger Son)

A 20-year-old male diagnosed with T-ALL at age 6 (2012) due to fever and ecchymosis.

#### Initial features

**CBC**: WBC 1.6×10^9^/L, Hb 84g/L, PLT 13×10^9^/L.

**Bone Marrow**: 51% blasts/immature lymphocytes.

**Immunophenotyping**: CD7/CD5/CD8/cCD3 positive, with myeloid crossover expression (CD13/CD33).

**Karyotyp**e: 46,XY[20].

**Diagnosis:** T-ALL(with myeloid crossover expression).

#### Treatment and outcome

**Induction Therapy** (VDLP regimen): vincristine 1.5 mg/m² (max 2 mg), iv, weekly, days 1, 8, 15, 22; daunorubicin 45 mg/m², iv, days 1-3; pegaspargase 2,500 IU/m², im, day 3, prednisone: 60 mg/m², po, days 1-28 (tapered after Day 14).

**Consolidation** (CAM + HD-MTX regimens):

CAM: cyclophosphamide 1,000 mg/m², iv, day 1; cytarabine:75 mg/m²,iv, days 1-4 & 8-11; mercaptopurine (6-MP) 60 mg/m², po, days 1-14;

High-Dose Methotrexate (HD-MTX): 5 g/m² iv over 24 hours (with leucovorin rescue).

**Intensification** (VDLD + EA regimens):

VDLD: vincristine 1.5 mg/m² (max 2 mg), iv, days 1, 8; daunorubicin 45 mg/m², iv, days 1, 8; pegaspargase 2,500 IU/m², im, day 3, dexamethasone: 10 mg/m², po, days 1-14.

EA: etoposide: 200 mg/m², iv, days 1, 4,8; cytarabine: 300 mg/m², iv, days 1, 4,8.

**Maintenance Therapy**: 6-MP 50 mg/m², po, daily, methotrexate: 20 mg/m², po/im, weekly.

**CNS Prophylaxis**: Triple Intrathecal Therapy (×20 doses): methotrexate 12 mg cytarabine 25 mg, dexamethasone 5 mg.

**Molecular Genetics**: Germline testing in 2023 confirmed the VHL c.385C>G mutation (VAF 49.5%), while excluding ASXL3/CCR7 variants. No somatic mutations were detected in the analyzed genes.

**Follow-Up**: He maintained 16-year disease-free survival (DFS) and overall survival (OS) (as of April, 2025).

## Discussion

This study reports a rare familial hematological pedigree with germline mutations in ASXL3, CCR7, and VHL, presenting heterogeneous phenotypes of AML, ITP, and T-ALL. Notably, only the VHL mutation was shared among all affected individuals, whereas ASXL3/CCR7 were restricted to the proband and one child (Patient 2). Given this, the VHL mutation appears to be the only shared genetic factor underlying all three hematological phenotypes (AML, ITP, and T-ALL), while ASXL3 and CCR7 may act as phenotype modifiers rather than core predisposition drivers. The proband (AML) progression likely resulted from the combined effects of genetic predisposition and acquired somatic events (e.g., BCOR truncation). The eldest son (ITP) inherited ASXL3 and CCR7 mutations, with acquired BCOR mutations exacerbating immune dysregulation.

This finding provides novel clinical evidence for the synergistic pathogenic mechanisms of multigene germline mutations and offers critical insights for genetic counseling and clinical management of familial hematological disorders.

### Genetic mechanisms

Studies have shown that the ASXL family can cooperatively regulate epigenetic modifications through interactions with BAP1, EZH2, and nuclear receptors; however, the specific mechanisms by which ASXL3 participates in and modulates epigenetic regulation remain unknown (6, 7). CCR7 dysfunction could disrupt lymphocyte homing, contributing to autoimmune features in ITP (8–10). The younger son (T-ALL) carried only the VHL mutation, where hypoxia signaling may disrupt hematopoietic stem cell homeostasis, promoting clonal expansion in T-ALL (2, 3, 11), a mechanism established in VHL-related solid tumors but less explored in lymphoid malignancies (12, 13).

The phenotypic heterogeneity underscores the threshold-dependent and cross-pathway interactions of multigene mutations. For example, ASXL3-mediated chromatin remodeling may amplify CCR7’s impact on lymphocyte migration, while VHL-related hypoxia could foster clonal evolution. The proband’s high acquired mutation burden (e.g., BCOR truncation) supports the “second hit” model, akin to DDX41 germline mutation pedigrees. The eldest son’s low-frequency BCOR mutation highlights how minor acquired events may breach phenotypic thresholds, emphasizing the need for dynamic monitoring in high-risk individuals. Whether ASXL3/CCR7 act as primary modifiers or secondary passengers requires functional validation.

### Clinical implications

Clinically, this pedigree underscores the need for stratified interventions. Carriers of ≥2 germline mutations should undergo biannual CBC, peripheral smear, and targeted sequencing to detect acquired variants. Treatment strategies should integrate mutation profiles: ASXL3-mutated patients may respond to hypomethylating agents (e.g., azacitidin and decitabine) (14, 15), while CCR7 dysfunction could benefit from JAK inhibitors (16, 17). The proband’s refractory thrombocytopenia, possibly linked to ASXL3-related megakaryocytic blockade, may require early epigenetic therapy. Genetic counseling should address the 50% inheritance risk and recommend preimplantation genetic diagnosis to prevent mutation transmission.

### Study limitations

This study is constrained by the small sample size of the pedigree and the incomplete confirmation of germline status for VHL, ASXL3, and CCR7, which hinders definitive conclusions about their pathogenic mechanisms. The absence of comprehensive germline validation also precludes clear distinction between inherited and *de novo* mutations. To address these gaps, future investigations integrating multicenter collaborative cohorts and induced pluripotent stem cell (iPSC)-based models are recommended, as they may help elucidate the synergistic regulatory networks of these mutations. Notably, despite these limitations, the pedigree described herein provides a valuable framework for optimizing genetic counseling strategies and advancing precision medicine approaches in familial hematological disorders.

## Conclusion

This pedigree provides compelling evidence that inherited VHL germline mutations confer susceptibility to a spectrum of hematologic malignancies (AML/ITP/T-ALL). Concurrently, the co-occurring ASXL3 and CCR7 variants are hypothesized to modulate phenotypic heterogeneity. These findings not only expand the known phenotypic repertoire of VHL-related disorders but also highlight the complex genotype-phenotype interplay in familial hematological malignancies, offering a foundation for personalized risk assessment and targeted therapeutic strategies in clinical genetics.

This pedigree demonstrates that VHL germline mutation can predispose to diverse hematologic malignancies, while ASXL3/CCR7 variants may direct phenotypic expression.

## Data Availability

The original contributions presented in the study are included in the article/supplementary material. Further inquiries can be directed to the corresponding authors.
